# Risk factors associated with mortality among patients with COVID-19 in the intensive care unit: prospective cohort study from the early pandemic phase in Brazil

**DOI:** 10.1016/j.clinsp.2026.100894

**Published:** 2026-02-28

**Authors:** Brena Ramos Athaydes, Mariane Vedovatti Monfardini, Priscila Marinho Abreu, Frederico Firme Figueira, Juliana Couto-Vieira, Roberta Ferreira Ventura Mendes, Rahyza Inacio Freire de Assis, Luiz Felipe Camporez, Priscilla de Aquino Martins, Liliana Cruz Spano, Sandra Ventorin von Zeidler

**Affiliations:** a Postgraduate Program in Biotechnology, Health Sciences Center, Universidade Federal do Espírito Santo (UFES), Vitória, ES, Brazil; b Hospital Estadual Jayme dos Santos Neves, Serra, ES, Brazil; c Postgraduate Program in Infectious Diseases, Health Sciences Center, Universidade Federal do Espírito Santo (UFES), Vitória, ES, Brazil

**Keywords:** SARS-CoV-2, Viral load, Intensive care units, Comorbidities, Mortality

## Abstract

**Objective:**

This prospective cohort study describes clinical, laboratory factors and virological factors associated with in-hospital mortality among 227 Intensive Care Unit (ICU) patients with SARS-CoV-2 infection admitted in Brazil during the pre-vaccination, pre-Omicron phase of the pandemic (September 2020 to March 2021).

**Methods:**

Clinical and laboratory data were extracted from medical records, and viral load was quantified by RT-qPCR Cycle threshold (Ct) values. Independent predictors of mortality were evaluated using multivariable Cox proportional hazards regression, and survival was analyzed with Kaplan-Meier curves.

**Results:**

Non-survivors were significantly older (*p* < 0.001) and more likely to have hypertension (*p* = 0.014) and chronic respiratory disease (*p* = 0.042). At admission, they exhibited higher leukocyte counts (*p* = 0.001), D-dimer (*p* = 0.028), and creatinine levels (*p* = 0.001), as well as more frequent tracheal intubation (*p* < 0.001). During hospitalization, non-survivors showed persistently elevated leukocyte counts and developed more complications, including acute kidney injury, septic shock, and cardiac arrest (*p* < 0.001). Viral load was higher in non-survivors (*p* = 0.026) and among those requiring intubation (*p* = 0.042) or with cardiovascular disease (*p* = 0.016). In the multivariable Cox model, only creatinine at admission remained independently associated with mortality (HR = 1.18; 95% CI: 1.00–1.39; *p* = 0.049). Survival analysis showed reduced survival among patients aged ≥ 65-years (*p* = 0.004) and those with chronic respiratory disease (*p* = 0.029).

**Conclusions:**

This study integrates clinical, laboratory, and viral load data to detail the early COVID-19 severity and mortality patterns in a Brazilian ICU.

## Highlights

• Elevated serum creatinine at ICU admission independently predicted in-hospital mortality.
• Viral load correlated with disease severity but was not an independent predictor after adjustment.
• Advanced age and chronic respiratory disease were associated with reduced survival.
• Non-survivors showed higher D-dimer, leukocyte counts, and greater multiorgan dysfunction.
• This study provides a historical record of early COVID-19 mortality in a Brazilian ICU.

## Introduction

The COVID-19 pandemic impacted healthcare systems worldwide, with particularly severe effects in developing countries such as Brazil. During the early phase (2020–2021), before the availability of vaccines or targeted therapies, critically ill patients required extensive respiratory and hemodynamic support and frequently progressed to high morbidity and mortality in Intensive Care Units (ICUs).^1^^,^^2^ By the end of 2021, Brazil had recorded more than 22 million confirmed cases and over 600,000 deaths, with Espírito Santo reporting approximately 600,000 cases and 13,000 deaths. To date, cumulative totals have risen to over 37 million cases and 700,000 deaths nationwide, with the Southeast region showing the highest hospitalization and mortality rates.^3^^,^^4^ These figures illustrate the magnitude of the pandemic and reflect the critical situation faced by hospitals during its initial phase, characterized by high ICU occupancy and severe clinical presentations.

Patients admitted to the ICU during this first phase commonly presented with respiratory failure and multiple organ dysfunction, often associated with risk factors such as advanced age, obesity, diabetes, and cardiovascular disease. Early in the pandemic, uncertainties persisted regarding how comorbidities and demographic factors influenced outcomes.^5^^,^^6^ At that time, SARS-CoV-2 viral load was increasingly recognized as a potential determinant of disease severity,^7^ although studies investigating this relationship with critical outcomes in critically ill patients were still limited and largely descriptive.^8^^,^^9^ The quantification of viral RNA through RT-qPCR Cycle threshold (Ct) values rapidly emerged as an early approach to explore whether viral burden influenced disease severity and clinical outcomes.^7^^,^^8^

This study aims to document how clinical, laboratory, and viral load parameters were associated with survival outcomes among critically ill COVID-19 patients during the pre-vaccination and pre-Omicron period in Brazil. Using prospectively collected data from a COVID-19 single-center referral hospital between September 2020 and March 2021, it provides a detailed historical record of the pandemic’s first wave in a developing-country setting, offering an integrated view of the biological and systemic factors that shaped patient survival before the emergence of viral variants, vaccination, and modern therapeutic protocols.

## Material and methods

### Patients and data collection

This prospective cohort study was conducted in accordance with the STROBE guidelines and followed the principles of the Declaration of Helsinki. It was approved by the Human Research Ethics Committee of the Health Sciences Center at the Federal University of Espírito Santo (Protocol n° 4.175.329). Written informed consent was obtained from all participants prior to their inclusion in the study. The study included 227 adult patients with confirmed SARS-CoV-2 infection who were hospitalized in the intensive care unit of Hospital Estadual Dr. Jayme Santos Neves (HEJSN), the referral hospital for COVID-19 in Espírito Santo. These patients were enrolled between September 2020 and March 2021 and were followed until their outcome.

Espírito Santo is the smallest state in the Southeast region, covering 46,074.4 km^2^, with a population of 3833,486 inhabitants.^10^ In 2021, COVID-19 accounted for 24.63 % of deaths from infectious diseases in the state; by 2022, this rate had dropped to 7.7 %, while cardiovascular diseases once again became the leading cause of death (26.9 %).^11^ Espírito Santo ranks ninth in Brazil for income equality and has the fifth-highest Human Development Index (HDI = 0.77).^12^ The COVID-19 pandemic significantly impacted the local economy, causing a 4.4 % contraction in real GDP in 2020 despite nominal growth. Besides that, the hospital network was expanded, and HEJSN became the second-largest hospital in Brazil in terms of COVID-19 patient care, offering up to 286 ICU beds between December 2020 and February 2021.^13^

SARS-CoV-2 infection was confirmed through Real-Time quantitative Polymerase Chain Reaction (RT-qPCR) testing of Nasal and Pharyngeal (NP) swabs. The samples, placed in viral transport medium,^14^ were kept at 4 °C in a cooler and transported to the Federal University of Espírito Santo laboratory. They were processed within 72 hours and subsequently stored at −80 °C until testing. Clinical and sociodemographic variables were recorded within 48 hours of ICU admission and no later than 10 days after symptom onset. Data on age, sex, comorbidities, symptoms, hospitalization duration, oxygen support, laboratory findings, and outcomes were prospectively collected from electronic medical records at different time points during hospitalization, based on the prescriptions and clinical notes of general practitioners and attending physicians.

### Viral RNA extraction and RT-QPCR

RNA was extracted from 200 µL of NP swab samples using the PureLink™ Viral RNA/DNA Mini Kit (Invitrogen™, Carlsbad, CA, USA), following the manufacturer’s instructions. The extracted RNA was eluted to a final volume of 50 µL.

RT-qPCR reactions were performed using specific primers and hydrolysis probes targeting two regions of the SARS-CoV-2 nucleocapsid gene (N1 and N2), along with an internal control (RNase P - RP), as recommended by the Centers for Disease Control and Prevention.^14^ The reactions were carried out using the TaqPath™ 1-Step RT-qPCR Master Mix (Thermo Fisher Scientific, Waltham, MA, USA) on a StepOnePlus™ PCR system (Applied Biosystems, Carlsbad, CA, USA). Negative and positive controls (2019-nCoV, IDT, Iowa, USA) were included for assay validation. A sample was considered positive if amplification of the N1 and N2 targets exceeded the predefined threshold of 0.2, with a Ct value below 40.

### Statistical analysis

Descriptive statistics were used to summarize demographic, clinical and laboratory characteristics. Patients were compared according to clinical outcome (death or discharge). Non-normally distributed numerical variables were expressed as medians and Interquartile Ranges (IQR) and compared using the Mann-Whitney *U* test. Categorical variables were analyzed using Pearson’s chi-square or Fisher’s exact test, as appropriate.

Viral load, expressed as RT-qPCR Ct values, was analyzed both as a continuous variable (Mann-Whitney *U* Test) and as a categorical variable, defined as high (Ct < 20), moderate (Ct 20–29.9), or low (Ct ≥ 30), using the Kruskal-Wallis Test. When applicable, pairwise comparisons were performed for multiple group analyses. Spearman’s rank correlation was used to assess associations between Ct values, clinical and laboratory parameters, and the time from symptom onset to sample collection.

The Kaplan-Meier method was used to estimate survival probabilities, and differences between groups were assessed using the log-rank test. Cox proportional hazards regression was used to identify independent factors associated with in-hospital mortality. Covariates with a *p*-value < 0.10 in the univariate analysis were considered potential candidates for the multivariate model. The final model included age (≥65-years), sex, hypertension, chronic respiratory disease, leukocyte count, D-dimer, and serum creatinine, while Ct value (viral load) and time from symptom onset to sample collection were entered as adjustment covariates. The proportional hazards assumption was verified using log–log survival plots. Adjusted Hazard Ratios (aHR) with 95 % Confidence Intervals (95 % CI) were reported, and statistical significance was defined as *p* < 0.05. All analyses were performed using SPSS version 20.0 (IBM Corp., Armonk, NY, USA).

## Results

Hospitalized COVID-19 patients who died were significantly older (median 69- vs. 59-years, *p* < 0.001) and had higher rates of hypertension (65.6 % vs. 50.5 %, *p* = 0.014), chronic respiratory disease (14.1 % vs. 5.1 %, *p* = 0.042), and were current or former smoking (46.2 % vs. 20.0 %, *p* = 0.003). Educational level differed, with more having only elementary education (51.6 % vs. 41.4 %) and fewer with higher education (2.3 % vs. 16.2 %, *p* = 0.001) among those who died. Survivors more often reported previous viral exposure, defined as contact with confirmed or suspected COVID-19 cases (52.5 % vs. 35.2 %, *p* = 0.011). No significant differences were observed in sex, race, residence, antibiotic use, or symptoms such as dyspnea, fever, or cough (Table 1). The complete list and comparison of symptoms and comorbidities are provided in Supplementary Table S1.

**Table 1:** Epidemiological and clinical characteristics.

Characteristic, n (%)	Hospital Discharge (n = 99)	Death (n = 128)	Total (n = 227)	*p*-value
*Age, years (median)*	59.0 (41.0–70.0)	69.0 (59.0–80.0)	65.0 (54.0–76.0)	**<0.001**^b^
*Male sex*	58 (58.6)	64 (50.0)	122 (53.7)	0.198
*Race*				0.886^a^
White	38 (38.4)	44 (34.4)	82 (36.1)	
Black	12 (12.1)	17 (13.3)	29 (12.6)	
Multiracial	46 (46.5)	64 (50.0)	110 (8.5)	
Other or unknown	3 (3.0)	3 (2.3)	6 (2.6)	
*Schooling*				**0.001**^a^
Elementary school	41 (41.4)	66 (51.6)	107(7.1)	
High school	35 (35.4)	39 (30.5)	74 (32.6)	
Higher education	16 (16.2)	3 (2.3)	19 (8.4)	
Illiterate	7 (7.1)	14 (10.9)	21 (9.3)	
*Place of residence*				0.876
Urban	82 (8.8)	105 (82.0)	187 (2.4)	
Rural area	17 (17.2)	23 (18.0)	40 (17.6)	
*Previous virus exposure*^c^	52 (52.5)	45 (35.2)	97 (42.7)	**0.011**
*Primary care*	67 (67.7)	79 (61.7)	146 (64.3)	0.207^a^
*Antibiotic use*	97 (98.0)	128 (100)	225 (99.1)	0.189^a^
*Symptoms and signs of infection*				
Dyspnea	90 (90.9)	108 (84.4)	198 (87.2)	0.245^a^
Desaturation	80 (80.8)	103 (80.5)	183 (80.6)	0.747^a^
Fever	49 (49.5)	58 (45.3)	107 (47.1)	0.751^a^
Cough	65 (65.7)	75 (58.6)	140 (61.7)	0.354^a^
Myalgia	24 (24.2)	28 (21.9)	52 (22.9)	0.675^a^
Diarrhea	11 (11.1)	19 (14.8)	30 (13.2)	0.581^a^
*Comorbidities*				
Hypertension	49 (50.5)	84 (65.6)	133 (58.6)	**0.014**
Obesity	39 (39.4)	43 (33.6)	82 (36.1)	0.404
Diabetes	35 (35.4)	48 (37.5)	83 (36.6)	0.743^a^
Chronic respiratory disease	5 (5.1)	18 (14.1)	23 (10.1)	**0.042**^a^
Chronic kidney failure	5 (5.1)	9 (7.0)	14 (6.2)	0.653^a^
Cardiovascular disease	11 (11.1)	23 (18.0)	34 (15.0)	0.290^a^
*Current of former smoker*	13/65 (20.0)	36/78 (46.2)	49/143 (34.3)	**0.003**
*Alcohol consumer*	1/63 (1.6)	2/71 (2.8)	3/134 (2.2)	0.440^a^
*Chronic-use medications*				0.104
Only 1 type	9 (9.1)	12 (9.4)	19 (9.3)	
2 types	15 (15.2)	14 (10.9)	29 (12.8)	
3 types	12 (12.1)	12 (9.4)	24 (10.6)	
4 or more/polypharmacy	14 (14.1)	34 (26.6)	48 (21.1)	
None	38 (38.4)	34 (26.6)	72 (31.7)	

Data are presented as mean (SD), n (%), or median (IQR). p-value defined by Pearson's Chi-Square Test.

a Fisher’s Exact Test

b Mann-Whitney *U* Test

c Previous viral exposure indicates contact with confirmed or suspected cases. Primary care refers to the patient’s place of initial care. History of tobacco use was missing for 84 patients, and alcohol use for 93-patients. Values are shown as n/n (%), with percentages calculated relative to the group total. Statistically significant *p*-values (*p* < 0.05) are shown in bold.

Significant differences in laboratory and clinical parameters at admission were found between the groups. Non-survivors had higher leukocyte (*p* = 0.001) and neutrophil counts (*p* = 0.002), as well as elevated D-dimer (*p* = 0.028) and creatinine levels (*p* = 0.001). Lower arterial pH values (*p* = 0.006) indicated more pronounced metabolic impairment in non-survivors (Table 2).

**Table 2:** Characteristics of the patients at baseline.

Characteristic, n (%)	Hospital Discharge (n = 99)	Death (n = 128)	Total (n = 227)	*p*-value
***Laboratory findings***				
White blood cell count, mm³	9500 (6600–13,000)	12,050 (7575–18,225)	10,600 (7400–15,700)	**0.001**^b^
Platelet count, per mm³	228,000 (178,000–297,000)	216,500 (164,250–280,750)	222,000 (169,000–289,000)	0.220^b^
Neutrophils (segs), mm³	81.0 (76.50–84.50)	82.0 (80.0–87.0)	82.0 (78.0–86.0)	**0.002**^b^
Neutrophils (bands), mm³	2.0 (2.0–3.0)	2.0 (2.0–3.0)	82.0 (78.0–86.0)	0.548^b^
*C-reactive protein,* mg/L	142.2 (94.55–223.85)	178.0 (111.45–249.40)	157.8 (107.3–240.45)	0.069^b^
*D-dimer,* mg/L	1.43 (0.84–3.45)	2.54 (1.06–6.28)	1.83 (0.94–3.84)	**0.028**^b^
*D-dimer concentration, n (%)*				**0.026**
≥ 1 × upper limit of normal	34 (34.3)	33 (25.8)	67 (29.5)	
≥ 3 × upper limit of normal	34 (34.3)	67 (52.3)	101 (44.5)	
*Creatinine,* mg/dL	0.88 (0.74–1.10)	1.09 (0.80–1.60)	1.0 (0.77–1.41)	**0.001**^b^
*Fever on admission*	7 (7.1)	5 (3.9)	12 (5.3)	0.363^a^
*Arterial pressure,* mmHg	135/81 (120/71–147/90)	130/80 (117/70–142/87)	130/80 (119/70–146/88)	0.058^b^
*Heart rate, bpm*	84 (73–96)	91 (78–106)	89 (76–100)	**0.004**^b^
*Gasometry*				
pH	7.40 (7.34–7.45)	7.38 (7.30–7.41)	7.38 (7.33–7.43)	**0.006**^b^
pO_2_	83.0 (67.3–114.0)	84.90 (65.35–106.75)	83.90 (67.0–107.0)	0.652^b^
***Oxygen support required***				**<0.001**^a^
Catheter/oxygen mask	72 (72.7)	72 (56.3)	144 (3.4)	
High-flow nasal cannula	1 (1.0)	0 (0.0)	1 (0.4)	
Non-invasive ventilation	5 (5.1)	2 (1.6)	7 (3.1)	
Tracheal intubation	19 (19.2)	54 (42.2)	73 (32.2)	
None (room air)	2 (2.0)	0 (0.0)	2 (0.9)	
***Radiologic findings***				
Chest CT performed	39 (39.4)	34 (26.6)	73 (32.2)	0.109^a^
Pulmonary involvement ≥ 50 %	29/39 (74.4)	27/34 (79.4)	56/73 (76.7)	0.114^a^
Ground glass opacity	38/39 (97.4)	31/34 (91.2)	69/73 (94.5)	**0.026**^a^

Data are presented as mean (SD), n (%), or median (IQR). The reference value for D-dimer is 0.5 mg/L. D-dimer data were missing or not analyzed for 46 patients (23 per group); median values exclude these cases. Radiologic findings are shown as total n/n (%), unless otherwise specified. CT, Computed Tomography. Percentages were calculated within each group. *p*-values were determined using Pearson’s Chi-Square test.

a Fisher’s Exact Test

b Mann-Whitney *U* Test. Catheter or oxygen mask indicates oxygen flow up to 15 L/min; high-flow nasal cannula, 20–60 L/min. Statistically significant *p*-values (<0.05) are shown in bold.

Regarding hemodynamic parameters, non-survivors also had higher heart rates at admission (*p* = 0.004) and required tracheal intubation more frequently (*p* < 0.001). Among imaging findings, approximately 30 % of patients underwent chest Computed Tomography (CT), and most of them showed > 50 % lung involvement, with ground-glass opacities observed in nearly all cases (Table 2).

During hospitalization, significant differences emerged between discharged and non-survivors regarding laboratory trends and ventilatory support needs (Table 3). Leukocyte counts remained consistently higher in those who died, with significantly more patients showing values > 15,000 mm^3^ across all three-time intervals analyzed (up to 7-days, 8–14 days, and 15–28 days; *p* < 0.005). Thrombocytopenia (platelets < 100,000 mm³) was more frequent in non-survivors during the first period (*p* = 0.008), with no significant differences thereafter. Oxygen therapy requirements differed significantly between survivors and non-survivors at all time points (*p* < 0.001). By day-7, nearly 90 % of non-survivors and about one-third of survivors had been intubated, a pattern that remained consistent through days-14 and -28. Detailed data are shown in Supplementary Table S2.

**Table 3:** Follow-up data on patients’ progression during hospitalization at 7-, 14- and 28-day intervals.

Parameter, n (%)	Interval	Hospital Discharge (n = 99)	Death (n = 128)	Total (n = 227)	*p*-value
*Leukocytosis (≥ 15,000/*mm³*)*	Day 1 to 7	31 (31.3)	91 (71.1)	122/135 (53.7)	**<0.001**^a^
	Day 8 to 14	26/65 (40.0)	72/97 (74.2)	98/162 (60.5)	**<0.001**^a^
	Day 15 to 28	13/38 (34.2)	38/53 (71.7)	51/91 (56.0)	**0.003**^a^
*Thrombocytopenia (< 100,000/*mm³*)*	Day 1 to 7	4 (4.0)	19 (14.8)	23 (10.1)	**0.008**
	Day 8 to 14	4/65 (6.2)	10/98 (10.2)	14/163 (8.6)	0.132
	Day 15 to 28	2/38 (5.3)	7/54 (13.0)	9/92 (9.8)	0.471^a^
*Invasive ventilation*	Day 1 to 7	34 (34.3)	115 (89.8)	149 (65.6)	**<0.001**^a^
	Day 8 to 14	30/72 (41.7)	92/99 (92.9)	122/171 (71.3)	**<0.001**
	Day 15 to 28	19/39 (48.7)	52/52 (100.0)	71/91 (78.0)	**<0.001**^a^

Results are expressed as total n/n (%) due to variations in patients’ length of stay, indicating the number of admissions during the period. For statistical analysis, leukocytosis and thrombocytopenia were analyzed by comparing the distribution of patients across the categories of high, low, and normal counts within each outcome group. Oxygen therapy comparisons were based on the four categories (catheter/oxygen mask, non-invasive ventilation, tracheal intubation, and room air). Oxygen therapy with invasive ventilation data reflect the worst-case scenario during each patient’s hospitalization. *p*-values were determined using Pearson’s Chi-Square test.

a Fisher’s Exact Test; statistically significant *p*-values (< 0.05) are shown in bold.

The clinical course differed significantly between groups, with complications markedly more frequent among non-survivors. Acute kidney injury, hemodialysis, hypotension, norepinephrine use, septic shock, and cardiac arrest were all significantly more common in the death group (*p* < 0.001). Septic shock occurred in nearly all deceased patients, versus 13 % of survivors. Hospital stay was also longer among non-survivors, with a higher proportion hospitalized for more than 14-days (*p* = 0.046), a result that may reflect variability within this group, as some patients died early during admission, while others remained hospitalized for extended periods (Table 4).

**Table 4:** Clinical evolution outcomes.

Characteristic, n (%)	Hospital discharge (n = 99)	Death (n = 128)	Total (n = 227)	*p*-value
*Complications*				
Acute kidney injury	14 (14.1)	94 (73.4)	108 (47.6)	**<0.001**
Hemodialysis	10 (10.1)	68 (53.1)	78 (34.4)	**<0.001**
Hypotension	21 (21.2)	109 (85.2)	130 (57.3)	**<0.001**
Use of noradrenaline	31 (31.3)	120 (93.8)	151 (66.5)	**<0.001**
Septic shock	13 (13.1)	98 (76.6)	111 (48.9)	**<0.001**
Cardiac arrest	2 (2.0)	32 (25.0)	34 (15.0)	**<0.001**^a^
*Length of stay*				**0.046**
Until 7 days	24 (24.2)	28 (21.9)	55 (22.9)	
8 to 14 days	32 (32.3)	46 (35.9)	78 (34.4)	
15 to 28 days	21 (21.2)	41 (33.0)	62 (27.3)	
More than 28 days	22 (22.2)	13 (10.2)	35 (15.4)	

Percentages were calculated within each group. *p*-values were obtained using Pearson’s Chi-Square test.

a Fisher’s Exact Test. Statistically significant *p*-values (< 0.05) are shown in bold.

**Fig. 1:**
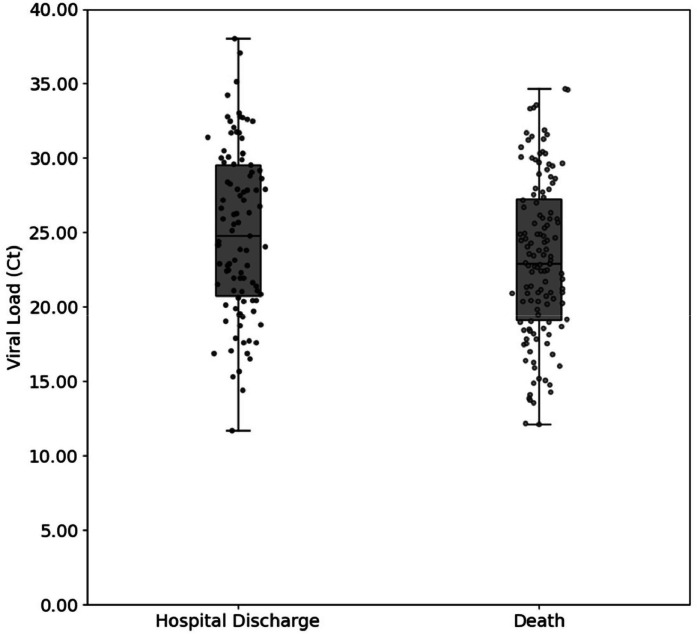
Distribution of viral load (Ct values) among hospitalized COVID-19 patients according to clinical outcome. Median Ct values were compared using the Mann-Whitney *U* test (*p* = 0.026), with dispersion of values illustrated.

Viral load, measured by Ct values, was significantly higher among patients who died, as indicated by lower Ct values (*p* = 0.026). The median Ct was 22.9 (IQR 19.1–27.3) in the death group, compared to 24.8 (IQR 20.6–29.6) among those who were discharged (Fig. 1).

Lower Ct values were also associated with oxygen therapy at admission (*p* = 0.033), particularly among patients requiring tracheal intubation than in those receiving oxygen via nasal cannula or mask (*p* = 0.042), and with cardiovascular disease (*p* = 0.016). Among those with cardiovascular disease, non-survivors had lower Ct values than discharged patients without the condition (*p* = 0.028).

When categorized as high (Ct < 20), moderate (Ct 20–29.9), or low (Ct ≥ 30), viral load showed significant associations. High viral load was more common in males (70.9 %) than females (29.1 %), while moderate viral load was more frequent among females (53.4 %) than males (46.6 %) (*p* = 0.010). Elevated creatinine (> 1.44 mg/dL) was also associated with high viral load (38.2 %) (*p* = 0.011).

Oxygen support differed by viral load (*p* = 0.012). Nasal cannula or oxygen mask was most common in the low viral load group (79.5 %) and least in the high viral load group (54.5 %), whereas tracheal intubation was more frequent in moderate (34.6 %) and high (40.0 %) viral load groups, suggesting greater severity. Cardiovascular disease was more prevalent in the high viral load group (27.3 %) compared to moderate (11.3 %) and low (10.3 %) groups (*p* = 0.027).

**Fig. 2:**
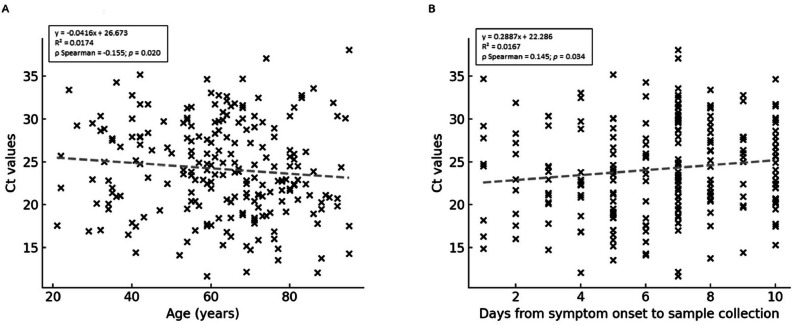
Spearman correlations between RT-qPCR Ct values and clinical variables in hospitalized COVID-19 patients. Scatter plots show correlations between Ct values and (A) age and (B) time from symptom onset to sample collection. Each point represents an individual patient. Correlation coefficients (rₛ) and *p*-values were calculated using Spearman’s rank test.

Spearman’s correlation showed a weak but significant inverse association between age and Ct values (*ρ* = -0.155, *p* = 0.020; R^2^ = 0.0174), indicating higher viral loads in older patients (Fig. 2A). Age also correlated positively with D-dimer (*ρ* = 0.170, *p* = 0.022). Ct values correlated positively with platelet count (*ρ* = 0.153, p = 0.021) and negatively with creatinine (*ρ* = -0.283, *p* < 0.001). A weak but significant positive correlation was also observed between time from symptom onset to sample collection and Ct values (*ρ* = 0.145, *p* = 0.034, R^2^ = 0.0167), indicating that samples collected later in the disease course tended to have higher Ct values (lower viral loads) (Fig. 2B). This finding supports the inclusion of time since symptom onset as an adjustment covariate in subsequent mortality analyses.

**Fig. 3:**
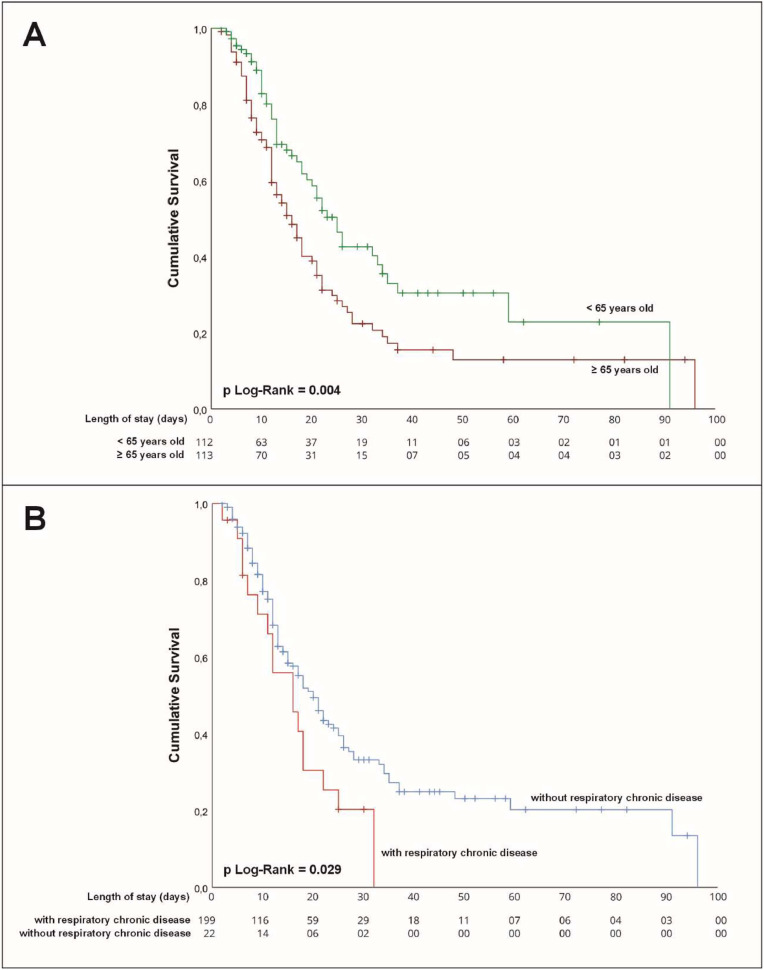
Kaplan-Meier survival curves for hospitalized COVID-19 patients. Censored cases represent hospital discharge, while uncensored cases represent in-hospital death. Survival curves are stratified by (A) age (< 65 vs. ≥ 65 years) and (B) presence of chronic respiratory disease. Log-rank test *p*-values are shown for each comparison. Tables below the curves indicate the number of patients at risk at each time point during hospitalization.

Survival curves differed significantly by age, with 65-years as the cutoff point based on ICU length of stay and death as the outcome (Fig. 3A). Patients under 65-years showed a longer median time to death compared to those aged 65 or older (25-days [IQR 20–30] vs. 16-days [IQR 13–19]; Log-Rank *p* = 0.004). Chronic respiratory disease was also associated with reduced survival, with patients presenting the condition showing a shorter median time to death than those without it (16-days [IQR 9–23] vs. 20-days [IQR 17–23]; Log-Rank *p* = 0.029) (Fig. 3B). No significant differences were observed according to sex (Log-Rank *p* = 0.929), cardiovascular disease (Log-Rank *p* = 0.589), or oxygen requirement at admission (Log-Rank *p* = 0.351).

In the univariate Cox regression model, age ≥ 65-years, chronic respiratory disease, and elevated creatinine levels were associated with an increased risk of in-hospital mortality (Table 5). Patients aged 65-years or older had a 1.66-fold higher risk of death compared with those under 65-years (HR = 1.66; 95 % CI: 1.16–2.37; *p* = 0.006), while the presence of chronic respiratory disease increased the risk by 72 % (HR = 1.72; 95 % CI: 1.04–2.85; *p* = 0.035). Higher serum creatinine levels were also associated with increased mortality (HR = 1.25; 95 % CI: 1.10–1.42; *p* = 0.001). No significant associations were found for sex, hypertension, leukocyte count, D-dimer, viral load (Ct), or time from symptom onset to sample collection (*p* > 0.05).

**Table 5:** Univariate and multivariate Cox regression analysis of predictors of in-hospital mortality among hospitalized COVID-19 patients.

Variable	CHR (95 % CI)	p-value	aHR (95 % CI)	*p*-value
*Age ≥65 years*	1.66 (1.16–2.37)	0.006	1.28 (0.80–2.04)	0.296
*Male sex*	1.02 (0.72–1.44)	0.931	1.06 (0.69–1.65)	0.784
*Hypertension*	1.22 (0.85–1.76)	0.289	1.32 (0.82–2.12)	0.253
*Chronic respiratory disease*	1.72 (1.04–2.85)	0.035	1.53 (0.78–2.98)	0.214
*Leukocytes (× 10³/*mm³*)*	1.00 (0.98–1.02)	0.656	1.01 (0.98–1.03)	0.556
*D-dimer (*mg/L*)*	1.00 (0.99–1.01)	0.418	1.00 (0.99–1.01)	0.341
*Creatinine (*mg/dL*)*	1.25 (1.10–1.42)	0.001	1.19 (1.01–1.40)	**0.041**
*Symptom onset to sample collection (days)*	0.96 (0.89–1.04)	0.326	0.99 (0.91–1.09)	0.877
*Ct value (viral load)*	0.99 (0.95–1.02)	0.379	0.99 (0.95–1.03)	0.602

Results from Cox proportional hazards regression showing crude (CHR) and adjusted (aHR) Hazard Ratios with 95 % Confidence Intervals (95 % CI). Statistically significant *p*-values (< 0.05) are shown in bold.

Variables with *p* < 0.10 in the univariate analysis (age, chronic respiratory disease, and creatinine), as well as clinically relevant covariates, were included in the multivariate Cox regression model. Ct values and symptom duration were also entered as adjustment covariates to control for potential confounding. After adjustment, only serum creatinine remained independently associated with mortality (aHR = 1.19; 95 % CI: 1.01–1.40; *p* = 0.041), indicating that for each 1 mg/dL increase in creatinine, the risk of death increased by approximately 19 %. None of the other variables retained statistical significance in the adjusted model (*p* > 0.05) (Table 5).

## Discussion

This study investigated factors associated with clinical progression and mortality among critically ill COVID-19 patients admitted to the ICU during the early, pre-vaccination and pre-Omicron phase of the pandemic (September 2020–March 2021). These data provide a detailed historical record of the first wave, when circulating variants, host immunity, and therapeutic protocols differed substantially from current standards. The authors identified several factors associated with mortality in this cohort, but serum creatinine at ICU admission emerged as the only independent predictor of in-hospital death, reinforcing the clinical relevance of early renal dysfunction as a marker of systemic disease severity.

Older age and pre-existing comorbidities were strongly associated with death as an outcome, as demonstrated by both clinical findings and survival analysis. Non-survivors were, on average, a decade older than survivors and had a higher prevalence of hypertension and chronic respiratory disease, conditions that reduce physiological reserve and impair vascular and pulmonary adaptation during severe infection.^15^, ^16^, ^17^ Age-related mechanisms such as immunosenescence, inflammaging, and endothelial dysfunction likely intensified systemic inflammation and delayed recovery.^18^ These factors probably contributed to their limited capacity to withstand the metabolic and hemodynamic stress of critical COVID-19, as shown by Kaplan-Meier curves indicating a shorter time to death among older adults and those with chronic respiratory disease.

The combined burden of chronic disease and age-related dysfunction appears to magnify the inflammatory cascade and compromise systemic resilience, explaining the higher proportion of severe complications and need for ventilatory support observed among non-survivors during hospitalization.^16^^,^^18^ This is consistent with a Brazilian study reporting a 41.3 % fatality rate among hospitalized patients, increasing to over 50 % in those with cardiovascular comorbidities and to 82.9 % among those requiring invasive ventilation,^19^ findings consistent with our observation that nearly 90 % of non-survivors were intubated by day-7, compared with less than one-third of survivors, a pattern also described by Fuenmayor-González et al.^20^

Non-survivors, especially those with cardiovascular or metabolic comorbidities, showed higher D-dimer and leukocyte counts at admission, consistent with the hyperinflammatory and prothrombotic profiles described in severe COVID-19.^21^, ^22^, ^23^ Metabolic acidosis and other laboratory abnormalities frequently coexisted with acute kidney injury, hypotension, and septic shock, complications developed during hospitalization, illustrating the extent of physiological stress and multi-organ dysfunction in these critically ill patients.^24^, ^25^, ^26^

Baseline viral load showed a similar pattern of association. Non-survivors presented with lower Ct values, indicating higher viral replication, which correlated with markers of disease severity such as oxygen support, renal dysfunction, and male sex, consistent with previous reports.^8^^,^^27^^,^^28^ However, viral load at admission should be interpreted with caution, as RNA levels fluctuate throughout the clinical course and typically peak within the first 10–12-days after symptom onset.^29^^,^^30^ When Ct values were adjusted for potential confounders in the multivariate Cox regression model, viral load did not remain an independent predictor of mortality, in line with previous studies showing that admission Ct values alone rarely predict outcomes such as intubation or death.^9^ Some investigations have also demonstrated that critically ill patients who clear the virus within two weeks, particularly those on invasive ventilation, experience better survival, whereas persistent viral detection correlates with worse prognosis, supporting a potential link between faster viral clearance and improved survival.^31^ These findings suggest that viral load at admission reflects the initial intensity of infection rather than determining survival outcomes directly, but outcomes ultimately depend on the host’s ability to regulate inflammation and limit secondary organ injury. In our study, viral quantification was performed only at baseline, which limits the ability to capture the dynamics of viral persistence or clearance during hospitalization and may have influenced the observed associations between Ct values and clinical outcomes.

Serum creatinine at ICU admission remained the only independent predictor of mortality in our multivariable Cox model, reinforcing its role as an early and integrative marker of systemic severity in COVID-19. Elevated creatinine reflects the convergence of key pathogenic mechanisms, such as cytokine-mediated inflammation, endothelial activation, and microvascular thrombosis, that compromise renal perfusion and filtration capacity.^32^^,^^33^ SARS-CoV-2 exhibits tropism for ACE2-expressing tubular epithelial cells and may cause direct cytopathic injury with mitochondrial impairment, podocyte damage, and collapsing glomerulopathy, leading to acute tubular necrosis and loss of filtration efficiency.^34^ Hemodynamic instability and perfusion deficits further compromise renal clearance, while infiltration of inflammatory cells expressing CD147 receptors promotes endothelial disruption and amplifies cytokine release, exacerbating renal inflammation and hypoperfusion.^35^ These interacting processes explain the renal dysfunction and creatinine elevation observed in severe COVID-19.

Clinically, even mild increases in serum creatinine at admission have been consistently associated with higher mortality across multiple cohorts. Russo et al.^36^ identified a threshold of ≥1.12 mg/dL linked to more than twice the odds of in-hospital death after adjustment for age and comorbidities. Similar admission cut-offs between 1.0–1.3 mg/dL have been confirmed as independent predictors of death in other studies.^37^^,^^38^ Our cohort showed a median creatinine level of 1.09 mg/dL (IQR 0.80–1.60) among non-survivors, aligning closely with these thresholds. Large observational datasets further indicate that early or peak increases in creatinine during hospitalization nearly double mortality risk, even after adjusting for confounders.^39^^,^^40^

Taken together, these findings emphasize that the clinical value of creatinine lies in its accessibility and ability to reflect systemic involvement. Measuring serum creatinine at admission may assist in early risk stratification, as reported by Mamven et al.,^39^ who also found that acute kidney injury at hospital presentation independently predicts mortality. This aligns with broader evidence indicating that renal involvement often represents a critical tipping point in the progression of systemic failure in COVID-19. The persistence of creatinine as a significant variable in our adjusted model reinforces that, during the early pandemic wave, when vaccination and advanced therapies were unavailable, renal impairment helped identify patients already progressing toward fatal systemic deterioration, underscoring its potential usefulness for early clinical assessment.

This study has several limitations. It was conducted in a single referral hospital in southeastern Brazil, which may limit generalizability. The findings must be interpreted within their temporal context, as the data reflect the early pandemic phase, before vaccination and the emergence of later variants, when both population immunity and treatment standards were markedly different. Differences in regional healthcare capacity and ICU management during this period may also have influenced mortality rates and the relative weight of risk factors. The high hospitalization rate led to the exclusion of some eligible patients due to missing or insufficient RT-PCR samples. As a retrospective analysis of electronic medical records, misclassification of certain clinical variables cannot be ruled out, although data collection followed a standardized protocol to ensure consistency. Mortality was assessed only for the in-hospital period, and post-discharge outcomes were not captured. Viral load was measured only once at hospital admission, limiting assessment of its temporal dynamics and precluding analysis of viral persistence or clearance during hospitalization, where a single-point quantification restricts the ability to infer temporal causality between viral load and survival. Although serum creatinine was identified as an independent predictor of mortality, the absence of pre-admission laboratory data limited the ability to distinguish acute from pre-existing renal dysfunction, despite the inclusion of chronic kidney disease status in the analysis. Likewise, other potentially relevant biomarkers, such as inflammatory cytokines, cardiac enzymes, and advanced renal markers like cystatin-C, were not systematically measured. Despite these limitations, this study provides a robust dataset from a COVID-19 referral center, contributing valuable historical evidence on disease severity patterns and mortality determinants during the pandemic’s early phase.

## Conclusions

In this historical cohort of critically ill COVID-19 patients from a referral hospital in the state of Espírito Santo, Brazil, admitted during the early pandemic phase, serum creatinine at ICU admission was the only factor independently associated with in-hospital mortality, indicating that early renal dysfunction mirrored the degree of systemic impairment and served as an integrative marker of disease severity. While age, comorbidities, and inflammatory burden contributed to adverse outcomes, their effects appeared largely mediated through multiorgan dysfunction. Although higher viral loads were observed among non-survivors and correlated with disease severity factors, viral quantification at admission alone did not independently predict survival, consistent with evidence that single-point measurements fail to capture the temporal dynamics of viral replication. Together, these findings provide a historical perspective on the biological and clinical mechanisms underlying early COVID-19 mortality, reflecting the disease profile in the pre-vaccination and pre-Omicron era.

## Abbreviations

ICU, Intensive Care Unit; RT-Qpcr, Reverse Transcription Quantitative Polymerase Chain Reaction; SARS-CoV-2, Severe Acute Respiratory Syndrome Coronavirus 2; Ct, Cycle threshold; COVID-19, Coronavirus Disease 2019; STROBE, Strengthening the Reporting of Observational Studies in Epidemiology; HEJSN, Hospital Estadual Dr. Jayme Santos Neves; HDI, Human Development Index; NP swabs, Nasal and Pharyngeal swabs; IQR, Interquartile Range; RNA, Ribonucleic Acid; RP, RNase P; CT, Computed Tomography; COPD, Chronic Obstructive Pulmonary Disease.

## Authors' contributions

All authors contributed to the study conception and design. Data curation was performed by Brena R. Athaydes, MSc; Priscila M. Abreu, PhD; Mariane M. V. Monfardini, PhD; and Roberta F. V. Mendes, PhD. Formal analysis and statistical interpretation were conducted by Brena R. Athaydes, MSc; and Priscila M. Abreu, PhD. Frederico F. Figueira, PhD; Juliana C. V. C. dos Santos, PhD; Rahyza I. F. de Assis, PhD; and Luiz F. Camporez collected samples and administered the questionnaires and consent forms. Funding acquisition was managed by Liliana C. Spano, PhD; and Sandra V. von Zeidler, PhD. All authors participated in the investigation and methodology of the study. Project administration was overseen by Liliana C. Spano, PhD, and Sandra V. von Zeidler, PhD, while supervision was provided by Sandra V. von Zeidler, PhD; Liliana C. Spano, PhD; and Priscilla A. Martins, MD. All authors contributed to data validation and visualization. Brena R. Athaydes drafted the original manuscript, with all authors contributing to its review and editing. All authors approved the final manuscript and agreed to be accountable for all aspects of the work.

## Funding

This work was supported by the Fundação de Amparo à Pesquisa e Inovação do Espírito Santo (FAPES) (grant number 283/2020) and the Coordenação de Aperfeiçoamento de Pessoal de Nível Superior (10.13039/501100002322CAPES) (grant number 692/2020). The funding sources had no role in the study design, data collection, analysis, interpretation of data, writing of the manuscript, or the decision to submit the article for publication.

## Data availability statement

The data that support the findings of this study are available from the corresponding author upon reasonable request, subject to ethical approval and data protection agreements established in informed consent.

## Declaration of competing interest

The authors declare no conflicts of interest.

## Appendix Supplementary materials

Image, application 1

## Footnotes

Supplementary material associated with this article can be found, in the online version, at doi:10.1016/j.clinsp.2026.100894.
