# Supplementary material for: Risk factors associated with mortality among patients with COVID-19 in the intensive care unit: prospective cohort study from the early pandemic phase in Brazil
Source: Clinics (Sao Paulo). 2026 Feb 28;81:100894. doi: 10.1016/j.clinsp.2026.100894 (PMC12961218; doi:10.1016/j.clinsp.2026.100894)
Supplement: Supplementary file 1 [file mmc1.docx]

**CLINICS-D-25-01401_ Supplementary Material**

**Supplementary Material**

**Table S1** Epidemiological and clinical characteristics (complete).

| **Characteristic, n (%)** | **Hospital Discharge**  **(n = 99)** | **Death**  **(n = 128)** | **Total**  **(n = 227)** | **p-value** |
| --- | --- | --- | --- | --- |
| *Symptoms and signs of infection* |  |  |  |  |
| Dyspnea | 90 (90.9) | 108 (84.4) | 198 (87.2) | 0.245^a^ |
| Oxygen desaturation | 80 (80.8) | 103 (80.5) | 183 (80.6) | 0.747^a^ |
| Fever | 49 (49.5) | 58 (45.3) | 107 (47.1) | 0.751^a^ |
| Cough | 65 (65.7) | 75 (58.6) | 140 (61.7) | 0.354^a^ |
| Odynophagia | 4 (4.0) | 6 (4.7) | 10 (4.4) | 0.834^a^ |
| Myalgia | 24 (24.2) | 28 (21.9) | 52 (22.9) | 0.675^a^ |
| Adynamia | 23 (23.2) | 23 (18.0) | 46 (20.3) | 0.566^a^ |
| Loss of taste/smell | 22 (22.2) | 15 (11.7) | 37 (16.3) | 0.094^a^ |
| Headache | 17 (17.2) | 22 (17.2) | 39 (17.2) | 0.784^a^ |
| Diarrhea | 11 (11.1) | 19 (14.8) | 30 (13.2) | 0.581^a^ |
| Nausea | 10 (10.1) | 7 (5.5) | 17 (7.5) | 0.336^a^ |
| Vomiting | 8 (8.1) | 7 (5.5) | 15 (6.6) | 0.512^a^ |
| Other | 20 (20.2) | 23 (18.0) | 43 (18.9) | 0.670 |
| *Body-mass index, kg/m²* | 28.6 (25.3 – 31.9) | 27.4 (25.0 – 31.2) | 27.8 (25.1 – 31.6) | 0.199^b^ |
| *Comorbidities* |  |  |  |  |
| Hypertension | 49 (50.5) | 84 (65.6) | 133 (58.6) | **0.014** |
| Obesity | 39 (39.4) | 43 (33.6) | 82 (36.1) | 0.404 |
| Diabetes | 35 (35.4) | 48 (37.5) | 83 (36.6) | 0.743^a^ |
| Chronic respiratory disease | 5 (5.1) | 18 (14.1) | 23 (10.1) | **0.042**^a^ |
| Chronic kidney failure | 5 (5.1) | 9 (7.0) | 14 (6.2) | 0.653^a^ |
| Cardiovascular disease | 11 (11.1) | 23 (18.0) | 34 (15.0) | 0.290^a^ |
| Autoimmune disease | 1 (1.0) | 4 (3.1) | 5 (2.2) | 0.504^a^ |
| Cancer | 0 | 6 (4.7) | 6 (2.6) | 0.053^a^ |
| Other | 25 (25.3) | 48 (37.5) | 73 (32.2) | 0.114^a^ |

Data are presented as mean (SD), n (%), or median (IQR). p-value defined by Pearson's Chi-Square Test.

^a^ Fisher’s Exact Test; ^b^ Mann-Whitney *U* Test; Values are shown as n/n (%), with percentages calculated relative to the group total. Statistically significant p-values (p < 0.05) are shown in bold.

**Table S2** Follow-up data on patient’s progression during hospitalization at 7-, 14- and 28-day intervals (complete).

| **Characteristic, n (%)** | **Hospital Discharge**  **(n = 99)** | **Death**  **(n = 128)** | **Total**  **(n = 227)** | **p-value** |
| --- | --- | --- | --- | --- |
| ***Laboratory findings*** |  |  |  |  |
| *White blood cell count, distribution per mm^3^* |  |  |  |  |
| Until day 7: |  |  |  | **<0.001**^a^ |
| ≥ 15,000 | 31 (31.3) | 91 (71.1) | 122/135 (53.7) |  |
| < 4,000 | 8 (8.1) | 2 (1.6) | 10 (4.4) |  |
| Day 8 to 14: |  |  |  | **<0.001**^a^ |
| ≥ 15,000 | 26/65 (40.0) | 72/97 (74.2) | 98/162 (60.5) |  |
| < 4,000 | 0/65 (0.0) | 1/97 (1.0) | 1/162 (0.6) |  |
| Day 15 to 28: |  |  |  | **0.003**^a^ |
| ≥ 15,000 | 13/38 (34.2) | 38/53 (71.7) | 51/91 (56.0) |  |
| < 4,000 | 3/38 (7.9) | 1/53 (1.9) | 4/91 (4.4) |  |
| *Platelet count, distribution < 100,000 per mm ^3^* |  |  |  |  |
| Until day 7: | 4 (4.0) | 19 (14.8) | 23 (10.1) | **0.008** |
| Day 8 to 14: | 4/65 (6.2) | 10/98 (10.2) | 14/163 (8.6) | 0.132 |
| Day 15 to 28: | 2/38 (5.3) | 7/54 (13.0) | 9/92 (9.8) | 0.471^a^ |
| ***Oxygen therapy*** |  |  |  |  |
| Until day 7: |  |  |  | **<0.001**^a^ |
| Catheter/oxygen mask | 37 (37.4) | 10 (7.8) | 47 (20.7) |  |
| Non-invasive ventilation | 25 (25.3) | 2 (1.6) | 27 (11.9) |  |
| Tracheal intubation | 34 (34.3) | 115 (89.8) | 149 (65.6) |  |
| None (room air) | 3 (3.0) | 1 (0.8) | 4 (1.8) |  |
| Day 8 to 14: |  |  |  | **<0.001** |
| Catheter/oxygen mask | 16/72 (22.2) | 4/99 (4.0) | 20/171 (11.7) |  |
| Non-invasive ventilation | 12/72 (16.7) | 3/99 (3.0) | 15/171 (8.8) |  |
| Tracheal intubation | 30/72 (41.7) | 92/99 (92.9) | 122/171 (71.3) |  |
| None (room air) | 14/72 (19.4) | 0/99 (0.0) | 14/171 (8.2) |  |
| Day 15 to 28: |  |  |  | **<0.001**^a^ |
| Catheter/oxygen mask | 6/39 (15.4) | 0/52 (0.0) | 6/91 (6.6) |  |
| Non-invasive ventilation | 6/39 (15.4) | 0/52 (0.0) | 6/91 (6.6) |  |
| Tracheal intubation | 19/39 (48.7) | 52/52 (100.0) | 71/91 (78.0) |  |
| None (room air) | 8/39 (20.5) | 0/52 (0.0) | 8/91 (8.8) |  |

Results are expressed as total n/n (%) due to variations in patients’ length of stay indicating the number of admissions during the period. Oxygen therapy data reflect the worst-case scenario during each patient’s hospitalization. Percentages were calculated within each group.

^a^ Fisher’s Exact Test; ^b^ Mann-Whitney *U* Test. Catheter/oxygen mask indicates oxygen flow up to 15 L/min. Statistically significant p-values (< 0.05) are shown in bold.
